# Dietary intake of fish, omega-3, omega-6 polyunsaturated fatty acids and vitamin D and the prevalence of psychotic-like symptoms in a cohort of 33 000 women from the general population

**DOI:** 10.1186/1471-244X-10-38

**Published:** 2010-05-26

**Authors:** Maria Hedelin, Marie Löf, Marita Olsson, Tommy Lewander, Björn Nilsson, Christina M Hultman, Elisabete Weiderpass

**Affiliations:** 1 Department of Neuroscience, Psychiatry, Ulleråker, Uppsala University, Uppsala, Sweden; 2 Department of Genetic Epidemiology, Samfundet Folkhälsan, Helsinki, Finland; 3 Department of Medical Epidemiology and Biostatistics, Karolinska Institutet, Stockholm, Sweden; 4 Mathematical Sciences, Chalmers University of Technology, Gothenburg, Sweden; 5 Cancer Registry of Norway, Oslo, and Department of Community Medicine, University of Tromsø, Tromsø, Norway

## Abstract

**Background:**

Low intake of fish, polyunsaturated fatty acids (PUFA) and vitamin D deficiency has been suggested to play a role in the development of schizophrenia. Our aim was to evaluate the association between the intake of different fish species, PUFA and vitamin D and the prevalence of psychotic-like symptoms in a population-based study among Swedish women.

**Methods:**

Dietary intake was estimated using a food frequency questionnaire among 33 623 women aged 30-49 years at enrolment (1991/92). Information on psychotic-like symptoms was derived from a follow-up questionnaire in the years 2002/03. Participants were classified into three predefined levels: low, middle and high frequency of symptoms. The association between diet and psychotic-like symptoms was summarized in terms of relative risks (RR) and corresponding 95% confidence intervals and was evaluated by energy-adjusted multinomial logistic regression.

**Results:**

18 411 women were classified as having a low level of psychotic-like symptoms, 14 395 as middle and 817 as having a high level. The risk of high level symptoms was 53% (95% CI, 30-69%) lower among women who ate fish 3-4 times per week compared to women who never ate fish. The risk was also lower for women with a high intake of omega-3 and omega-6 PUFA compared to women with a lower intake of these fatty acids. The effect was most pronounced for omega-6 PUFAs. The RR comparing the highest to the lowest quartile of omega-6 PUFAs intake was 0.78 (95% CI, 0.64-0.97). The associations were J-shaped with the strongest reduced risk for an intermediate intake of fish or PUFA. For fatty fish (herring/mackerel, salmon-type fish), the strongest inverse association was found for an intermediate intake (RR: 0.81, 95% CI, 0.66-0.98), whereas a high intake of fatty fish was associated with an increased risk of psychotic-like symptoms (RR: 1.90, 95% CI, 1.34-2.70). Women in the highest compared with the lowest quartile of vitamin D consumption experienced a 37% (95% CI, 22-50%) lower risk of psychotic-like symptoms.

**Conclusion:**

Our findings raise a possibility that adult women with a high intake of fish, omega-3 or omega-6 PUFA and vitamin D have a lower rate of psychotic-like symptoms.

## Background

Even though psychoses are relatively rare, between 5-15% of the general population has been estimated to report single schizophrenia-like symptoms like delusions, magical thinking, and hearing internal voices at some point in their lifetime [1-3]. The biological mechanisms underlying the etiology of schizophrenia and psychotic symptoms are largely unknown. Genetic constitution is important [4], but environmental factors like an unhealthy lifestyle with a poor diet may be involved [5,6].

Schizophrenia in adulthood is often preceded by milder symptoms and delusions during adolescence. The typical age of onset for schizophrenia is early adulthood (20-25 years of age). Expression of psychotic symptoms in populations is continuous and characterized by differing levels of severity and persistence [7]. Meta-analysis [8] and prospective follow-up studies indicates that up to 75-90% of developmental psychotic experiences are transitory. Persistence and clinical relevant impairment may be related to a family history of schizophrenia and environmental risk factors that might interact with the genetic risk. Self-reported psychotic experiences in the general population may represent the developmental expression of population genetic risk for psychosis [9].

Low maternal fish and seafood consumption during pregnancy is reported to increase the risk for a low IQ and suboptimal neuro-developmental outcomes in childhood [10], factors that in turn are associated with an increased risk for adult mental disorders like schizophrenia [11]. A recent meta-analysis found a latitude related increase in schizophrenia prevalence that was greater for groups with low fish consumption [12]. Fatty fish is a rich dietary source of essential fatty acids and vitamin D, both of which could be implicated in the development of schizophrenia.

For instance, it has been proposed that aberration in metabolism of phospholipids could be a biochemical basis for psychiatric disorders [13]. Neuronal membranes are largely made up of phospholipids, and the brain phospholipids are rich in polyunsaturated fatty acids (PUFA). The main groups of PUFA are omega-6 and omega-3 fatty acids, of which some need to be supplied through the diet. Eicosapentaenoic acid (EPA) and docosahexaenoic acid (DHA) belong to the omega-3 family, and are mainly found in fatty fish. Although, only hypothesis generating, two ecological studies support the hypothesis by reporting the ratio of saturated fat to PUFA in the diet is a strong predictor of schizophrenia outcome, measured as according to either clinical or to social variables [6,14]. Also, the dietary intake of PUFA was negatively correlated with the severity of psychotic symptoms in patients with schizophrenia [15]. Lower levels of PUFA have been found in brain content, red blood cells and skin fibroblast among patients with schizophrenia, compared with a healthy population [13,16]. Results from a review of several randomized clinical trials of PUFA treatment of schizophrenia were inconclusive, although, it seems that supplementation of especially EPA to these patients may have a positive effect on their schizophrenic symptoms [17].

Furthermore, it has been hypothesized that prenatal vitamin D deficiency is a risk factor for schizophrenia, supported by the role of this vitamin in cell growth and differentiation, the excess of winter births in schizophrenia (a period when vitamin D levels are low), and increased births of pre-schizophrenic subjects in urban areas, where vitamin D deficiency is higher [12,18].

However, to our knowledge, no study has investigated the association between dietary intake of fish, omega-3 and omega-6 PUFA or vitamin D and the risk of having positive psychotic symptoms in the general adult population.

The main purpose of the present study was to evaluate the association between the dietary intake of different fish species, the dietary intake of PUFAs (omega-3 and omega-6) and vitamin D and the prevalence of positive psychotic-like symptoms in a population-based study among Swedish women.

## Methods

### Study population

Women aged 30-49 years, residing in the Uppsala Health Care Region in Sweden during 1991 and 1992, form the source population for this study. Of this source population, 96 000 women were randomly selected from four age strata (30-34, 35-39, 40-44 and 45-49 years) and were invited to participate in the Swedish component of the Scandinavian Women's Lifestyle and Health Cohort [19,20]. The women were asked to fill in a paper questionnaire, including a food frequency questionnaire (FFQ), and levels of fish, PUFAs and vitamin D intake were evaluated. Of those invited, over half decided to participate. Thus, 49 261 returned the questionnaires and were enrolled in the study.

In 2002/2003 a follow-up study was initiated, and women who had responded to the 1991/1992 questionnaire and who were alive and living in Sweden in October 2002 were contacted. Since 1991/1992, 688 women were deceased, and 491 women had emigrated. 47 859 women were invited to complete a web-based questionnaire, and non-responders received a paper questionnaire. The overall response rate was 72%, and 34 415 answered the follow-up questionnaire and levels of psychotic-like symptoms was measured (outcome under study). A detailed description of the follow-up study and exposure assessment has been described elsewhere [21]. The Swedish Data Inspection Board and the regional Ethical Committee approved the study.

### Ratings of positive psychotic-like symptoms

The follow-up questionnaire contained 20 questions on psychotic-like symptoms, (Additional file 1), constituting the positive symptoms of the CAPE (Community Assessment of Psychic Experiences) questionnaire, a modified version of the Peters et al. Delusions Inventory, [PDI; [22]]; which is based on the 9^th ^edition of the Present State Examination [23]. The questions are styled in a 'Do you ever feel/think' fashion in order to study continuous experiences during life-time. The CAPE tool has proven to be a stable, valid and reliable self-report instrument for the measurement of psychotic-like experiences in the general population based on cross-validation with interview-based data [24,25]. The questions were translated from English into Swedish and back-translated to increase fidelity to the original scale. Two independent professional translators did the back-translation, and the consensus version was tested in a pilot study with 50 subjects.

From the responses to the questions on positive psychotic-like symptoms, a variable was created by categorizing women into three groups (Additional file 1). The "low level symptoms group" included women with no or few experiences of psychotic-like symptoms (≤3 "sometimes" and no "almost always" and "often" answers to any of the questions). The "high level symptoms group" included women with frequent experiences of psychotic-like symptoms (≥3 "almost always" or "often" answers). The "middle level symptoms group" was defined as participants not included in the low level or high level groups.

### Diet and lifestyle exposure assessment

The self-administered questionnaire in the parent study assessed lifestyle variables (smoking history, alcoholic drinking), anthropometry (height, weight, body mass index, BMI), medical history (previous diagnosis of major diseases) and average intake of foods and beverages [19]. Dietary habits during the 6 months preceding the woman's enrolment in the study were ascertained through a validated FFQ that covered the frequency of consumption and quantity of about eighty food items and beverages [26]. The validity of the fat estimates from the FFQ assessed using Pearson correlation coefficients between FFQ data and estimates derived from weighed food records varied between r = 0.4 and r = 0.5. The validity of PUFA estimated by means of the FFQ was r = 0.5 in comparison to adipose tissue composition [26]. As part of the FFQ, the participants reported how often, on average, they ate salmon-type fish (Baltic herring, herring, or mackerel), white fish (cod, saithe, or pike), caviar, or shellfish (e.g., shrimp): never-seldom, 1-3 times/month, 1 time/week, 2 times/week, 3-4 times/week, 5-6 times/week, 1 time/day, 2 times/day or 3 times/day.

The average intake of food items from the FFQ were converted into average intake of energy and nutrients by linkage to the database of nutrients created by the Swedish National Food Administration [27]. To estimate the total intake of omega-3 fatty acids, we summarized the intake of α-linolenic, EPA, DHA and docosapentaenoic acids (DPA). We combined EPA, DHA and DPA to estimate the total intake of marine fatty acids. To estimate the total intake of omega-6 fatty acids, we combined the intake of arachidonic and linoleic acids. We are aware that some arachidonic acid (AA) could be found in fish [27], however, we choose to include those fatty acids that are dominating in fatty fish into the variable "marine fatty acids".

AA exists in limited levels in liver, meat and eggs, but can be metabolized in humans from other fatty acids in the omega-6 fatty acid family. Linoleic acid is the parent fatty acid of the omega-6 family, and the main source in a typical Swedish diet is vegetable oil (such as corn oil, sunflower oil, soy oil, rapeseed oil and margarine). α-linolenic acid, the parent fatty acid of the omega-3 family can, to a limited extent, be converted into EPA, DPA and DHA. Conventional dietary sources of α-linolenic acid are rapeseed oil, soy oil, dark green leafy vegetables, flax seed, walnuts and soy beans. EPA and DHA are mainly found in fatty fish, with levels that vary by the species of the fish, environmental factors and geographic area [27]. However, we were not able take environmental factors and geographic area into account, because the study questionnaire did not assess the origin of fish, such as the Baltic Sea or the Atlantic Ocean.

### Statistical methods

Among the 34 415 women included in the study, we had information on dietary intake (parent study) and psychotic-like symptoms for 34 310 women (follow-up study). Participants with an energy intake outside the first (2261 kJ/d) and 99th (12 335 kJ/d) percentiles were excluded from the analysis (n = 687). Thus, a total of 33 623 women were included in the analysis.

Baseline characteristics between the low and the high level group of psychotic-like symptoms were compared using a two-sided t-test for equal means for continuous, normally distributed variables and and χ^2^-test for categorical variables. Non-normally distributed variables were log-transformed to normalize the distribution.

The association between fish, fatty acids or vitamin D and psychotic-like symptoms was summarized in terms of relative risk ratios (RRRs) and corresponding 95% confidence intervals, and it was evaluated by energy-adjusted multinomial logistic regression (polytomous logistic regression), for example: RRR = P(y = high level group; fish intake >2/week)/P(y = low level group; fish intake >2/week)/P(y = high level group; no fish intake)/P(y = low level group; no fish intake). The category "low level symptoms group" (no psychotic-like symptoms) was used as the reference group. The estimated associations given by a multinomial logistic regression are relative risk ratios (RRR). For simplicity of language we abbreviated 'relative risk ratio' to 'relative risk' (RR). Since, the outcome status (namely psychotic symptoms) among participants was unknown at study entry we cannot draw any conclusions about causality, only about the existence of associations (negative or positive). Based on the hypothesis under study we interpreted the RR<1 as a negative association (for simplicity we refer to it from now on "decreased risk") and RR>1 as a positive association (for simplicity we refer to it from now on as "increased risk").

Nutrient density was calculated by dividing the estimated intake of fatty acids, vitamin D and other nutrients by the total energy intake (i.e., the multivariate nutrient density model) [28]. The intake of fatty acids and vitamin D was categorized into quartiles, with the lowest quartile as the reference category for comparison. The intake of individual seafood items was grouped into four categories (none, 1-3 times per month, 1 time per week and 2 times per week or more). The total intake of all fish and seafood was grouped into six categories (none, 1-3 times per month, once per week, twice per week, 3-4 times per week and 5 times per week or more).

Age- and energy-adjusted models (with age in 5-year intervals and total energy intake as a continuous variable) were fitted, as well as models adjusted for additional potential confounders, including categories of BMI (< 25, 25-29.9, 30 or more), level of education (0 to 10 years, 10-13 years, 13+ years), country of birth (Nordic countries or other countries), smoking (yes, no), and intake of selected food groups and nutrient densities (fish other than the main exposure of interest, meat, dairy products, vegetables, fruits, cereals, refined sugar, alcohol, fatty acids other than the main exposure of interest, retinol, and vitamins A, B6, and B12), categorized into quartiles, as well as rheumatoid arthritis (yes, no), gluten intolerance (yes, no), diabetes (yes, no), intake of multivitamin supplements (never, occasional, regular). In an additional analysis of the association between alcohol intake and psychotic-like symptoms, we categorized women into never drinkers or drinkers, and used drinkers as the reference category. The selection of covariates included in the final multivariate models was based on proportional (≥10%) change in β-coefficients and previous subject matter knowledge. We initially tested all covariates, and those included in the final models were considered to be important confounding factors for the relation between the main exposure and psychotic-like symptoms. They are listed in the table footnotes. We decided not to include dietary Vitamin D estimates in the final multivariate models as fish is a rich source of vitamin D and could account for some of the effect of fish on our outcome. Additionally, there is a high correlation between vitamin D and omega-3 fatty acids (correlation = 0.77). Statistical analyses were performed using the STATA version 10.0

## Results

### Characteristics of study participants

Baseline characteristics of the study participants are presented in Table 1. The women in the high level group of psychotic-like symptoms were significantly younger, had a higher prevalence of overweight and obesity and were less educated than women with less or no experience of psychotic-like symptoms. Furthermore, a higher proportion of the women in the middle and high level symptoms groups had grown up outside of the Nordic countries than women in the low level symptoms group of psychotic-like symptoms. The RR comparing growing up outside of the Nordic countries for the middle group and the high level group compared with the low level group was 1.7 (95% CI, 1.5-2.0) and 5.6 (95% CI, 4.0-6.8), respectively. Women with no or few psychotic-like symptoms smoked less than women in the middle group and the high level group. The RR comparing ever smokers to never smokers, for the middle group and the high level group compared with the low level group was 1.2 (95% CI, 1.1-1.3) and 1.5 (95% CI, 1.3-1.7), respectively. Women in the high level group were more likely to be never drinkers than women in the low level group, the multivariate RR, adjusted for smoking, BMI, education and country of birth, was 1.5 (95% CI, 1.2-1.8). There was no association between alcohol intake and psychotic symptoms among women in the middle and low psychotic-like symptoms groups (data not shown). The remaining dietary intake of different food items and specific nutrients were similar among the three groups of women with different levels of positive psychotic-like symptoms (Table 1).

**Table 1:** Selected baseline characteristics by categories of psychotic-like symptoms, of 33 623 participants with questionnaire data in the women's lifestyle and health study

	Positive psychotic-like symptoms^a^		
	
	Low level group	Middle level group	High level group
	N = 18 411	N = 14 395	N = 817
Characteristics	(55%)	(43%)	(2.4%)
Age ^b^, years, mean (SD)	52 (6)	51 (6)	50 (6)
BMI, kg/m^2^, mean (SD)	23 (3.4)	23 (3.8)	24 (4.0)
**BMI, kg/m^2^, No. (%)**			
< 25 normal weight	13 381 (73)	10 030 (70)	506 (62)
25-29.9 overweight	3 697 (20)	3005 (21)	198 (24)
≥ 30 obese	770 (4)	857 (6)	71 (9)
missing	563 (3)	503 (3)	42 (5)
**Education, No. (%)**			
0-10 years	4 853 (26)	3 829 (27)	235 (29)
11-13 years	6 899 (37)	5 845 (41)	336 (41)
over 13 years	6 342 (34)	4 463 (31)	227 (28)
missing	317 (2)	258 (2)	19 (2)
**Country of birth, No. (%)**			
northern countries	18 060 (98)	13 942 (97)	744 (91)
other countries	318 (2)	427 (3)	68 (8)
missing	33 (0.2)	26 (0.2)	5 (0.6)
**Smokers, No. (%)**			
never	8 111 (44)	5 702 (40)	281 (35)
ever	10 250 (56)	8 656 (60)	534 (65)
missing	50 (0.3)	37 (0.3)	2 (0.2)
**Alcohol intake, No. (%)**			
ever	16 258 (88)	12 614 (88)	668 (82)
never	2 153 (12)	1 781 (12)	149 (18)
**Dietary intake, g/day, median (5-95%) of:**			
fatty fish	7 (0-21)	7 (0-21)	7 (0-28)
meat	76 (26-144)	78 (22-149)	77 (3-167)
dairy products	330 (20-831)	331 (14-850)	320 (4-857)
vegetables	79 (22-177)	79 (21-186)	80 (19-214)
fruit	107 (15-315)	106 (11-311)	102 (7-337)
cereals	125 (47-280)	129 (47-288)	129 (41-299)
saccharides (sugar)	20 (7-44)	20 (7-45)	21 (6-51)
alcohol	2.5 (0-11)	2.4 (0-11)	1.8 (0-12)
marine fatty acids^c^	0.27 (0.07-0.6)	0.27 (0.06-0.6)	0.26 (0.05-0.7)
omega-3 fatty acids^d^	1.3 (0.6-2)	1.3 (0.6-2)	1.2 (0.6-2)
omega-6 fatty acids^e^	4.8 (2-8)	4.8 (2-8)	4.7 (2-8)
vitamin D (μg/day)	4.0 (1.8-7.1)	3.9 (1.7-7.2)	3.8 (1.4-7.1)
Total energy intake, kJ/day, mean (SD)	6 509 (1 801)	6 626 (1 886)	6 637 (1 986)
**Proportion of total energy intake, % from:**			
• fat	31	31	31
• protein	16	16	16
• carbohydrate	51	51	51
• alcohol	1	1	1

^a ^Participants categorized into levels of psychotic-like symptoms; low, middle, high (see methods section)

^b ^Age at the completeness of the follow-up questionnaire

^c ^Sum of eicosapentaenoic acid (EPA), docosahexaenoic acid (DHA) and docosapentaenoic acid

^d ^Sum of eicosapentaenoic acid (EPA), docosahexaenoic acid (DHA), docosapentaenoic acid and α-linolenic acid

^e ^Sum of arachidonic and linoleic acids

### Dietary intake of fish and risk of positive psychotic-like symptoms

**Figure 1:**
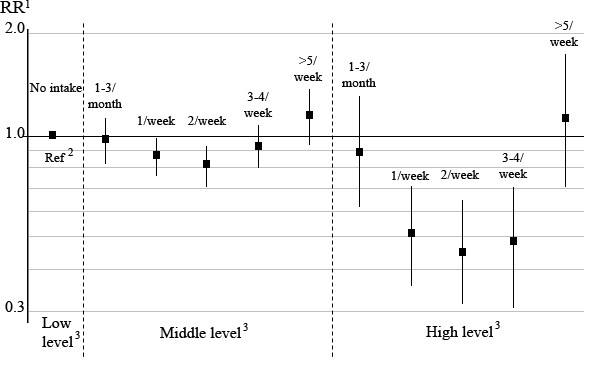
**Relative risk of positive psychotic-like symptoms in relation to estimated dietary intake of all fish and seafood ^4^**. ^1^Relative risk (RR) and 95% confidence interval evaluated by multinomial logistic regression, adjusted for age, total energy intake and dietary intake of vegetables, vitamin B12 and alcohol. ^2 ^The group with no or low level of psychotic symptoms was used as reference group. ^3 ^Participants categorized into levels; no/low, middle, high frequency of psychotic-like symptoms. ^4 ^Total intake of all fish and seafood were grouped into six categories; none, 1-3 times per month, once per week, twice per week, 3-4 times per week and 5 times per week or more.

The risk of positive psychotic-like symptoms in relation to estimated dietary intake of fish is shown in Table 2. The risk of belonging to the high or middle psychotic-like symptom group compared to the low level group was significantly lower among women with a high intake of white fish (cod/saithe/pike) or total fish and seafood products. For example, after multivariate adjustment, the risk of high level psychotic-like symptoms was 53% (95% CI, 30-69%) lower for women who ate all types of fish and seafood 3-4 times per week, and 55% (95% CI, 46-68%) lower for women who ate white fish two times per week, compared to women who never ate fish and seafood or white fish (Figure 1). However, there was a J-shaped association between psychotic-like symptoms and fatty fish (herring/mackerel and salmon-type fish) with the strongest inverse association for intermediate dietary intake (RR: 0.81, 95% CI, 0.66-0.98), whereas a high intake (RR: 1.90, 95% CI, 1.34-2.70) of fatty fish was associated with an increased risk of high level psychotic-like symptoms. In the high level symptoms group compared with the low level symptoms group, the intake of shellfish 1-3 times per month was associated with a reduced risk, whereas the intake of shellfish more than three times per week was associated with an increased risk. The latter result did not remain significant after multivariate adjustment (Table 2).

**Table 2:** Relative risk of positive psychotic-like symptoms in relation to estimated dietary intake of fish

Dietary intake	Positive psychotic-like symptoms^a^											
	
	Low level group		Middle level group					High level group				
	
				Energy adjusted		Multivariate^b^			Energy adjusted		Multivariate^b^	
	No.		No.	RR	95% CI	RR	95% CI	No.	RR	95% CI	RR	95% CI
**Salmon-type fish and herring/mackerel, frequency**												
never	5 027	Ref.	4 090					252				
1-3 per month	5 962		4 547	0.93	0.88-0.98	0.99^c^	0.93-1.05	216	0.72	0.60-0.86	0.81^c^	0.66-0.98
1 per week	6 381		4 727	0.90	0.85-0.95	0.99^c^	0.93-1.05	248	0.76	0.64-0.90	0.92^c^	0.75-1.12
≥2 per week	494		526	1.27	1.12-1.43	1.32^c^	1.15-1.51	48	1.74	1.26-2.39	1.90^c^	1.34-2.70
**Cod/saithe/pike, frequency**												
never	1 289	Ref.	1 136					96				
1-3 per month	7 009		5 311	0.85	0.78-0.93	0.89^d^	0.82-0.98	322	0.62	0.49-0.78	0.70^d^	0.55-0.89
1 per week	8 096		6 185	0.84	0.77-0.92	0.87^d^	0.80-0.95	270	0.43	0.34-0.55	0.46^d^	0.36-0.59
≥2 per week	1 470		1 258	0.93	0.83-1.04	0.90^d^	0.81-1.01	76	0.69	0.50-0.93	0.57^d^	0.41-0.79
**Shellfish, frequency**												
never	4 540	Ref.	3 815					243				
1-3 per month	11 075		8 219	0.88	0.84-0.93	0.90^e^	0.85-0.95	412	0.70	0.59-0.82	0.78^e^	0.66-0.93
1 per week	1 893		1 453	0.91	0.84-0.99	0.92^e^	0.84-1.00	78	0.77	0.59-1.00	0.84^e^	0.64-1.11
≥2 per week	356		403	1.34	1.15-1.55	1.28^e^	1.09-1.49	31	1.70	1.16-2.48	1.42^e^	0.94-2.14
**All fish and seafood, frequency**												
never	533	Ref.	476					44				
1-3 per month	1 131		996	0.96	0.82-1.11	0.96 f	0.83-1.12	84	0.87	0.60-1.27	0.89 f	0.61-1.31
1 per week	7 025		5 450	0.83	0.73-0.95	0.86 f	0.76-0.89	290	0.48	0.34-0.67	0.51 f	0.36-0.71
2 per week	7 215		5 261	0.77	0.68-0.88	0.82 f	0.71-0.93	264	0.42	0.30-0.58	0.45 f	0.32-0.64
3-4 per week	1 987		1 668	0.88	0.77-1.02	0.93 f	0.80-1.07	80	0.45	0.31-0.66	0.47 f	0.31-0.70
>5 per week	520		541	1.09	0.92-1.30	1.13 f	0.94-1.35	55	1.19	0.78-1.81	1.12 f	0.72-1.74

^a ^Participants categorized into levels of psychotic-like symptoms; low, middle, high (see methods section)

^b ^In the multivariate analysis presented, we initially tested the effects of adjusting also for BMI, level of education, country of birth, smoking, dietary intake of meat, dairy products, fruits, cereals and refined sugar, rheumatoid arthritis, gluten intolerance, diabetes and multivitamin supplement. However, none of these covariates change the estimates substantially, and was therefore not included in the final multivariate model

^c ^Adjusted for age, total energy intake and dietary intake of vegetables, vitamin B12, alcohol, cod-type fish and shellfish

^d ^Adjusted for age, total energy intake and dietary intake of vegetables, vitamin B12, alcohol, salmon-type fish and shellfish

^e ^Adjusted for age, total energy intake and dietary intake of vegetables, vitamin B12, alcohol, salmon-type fish and cod-type fish

^f ^Adjusted for age, total energy intake and dietary intake of vegetables, vitamin B12 and alcohol

### Dietary intake of omega-3, omega-6 fatty acids, vitamin D and risk of positive psychotic-like symptoms

The relative risk of positive psychotic-like symptoms by the level of fatty acids intake is shown in Table 3. After multivariate adjustment, the intake of omega-6 fatty acids was significantly associated with a decreased relative risk of psychotic-like symptoms. In women belonging to the high level symptoms group, the RRs with increasing quartiles of omega-6 intake were: 0.67 (95% CI, 0.55-0.82), 0.66 (95% CI, 0.54-0.81), 0.78 (95% CI, 0.64-0.97). The results for omega-3 fatty acid and marine fatty acids (EPA, DHA) had a similar pattern, indicating a reduced risk of psychotic-like symptoms among women with intermediate levels of intake. After multivariate adjustment, the risk of high level psychotic-like symptoms for intake of omega-3 or marine fatty acids was 24% lower in the third quartile compared to the lowest.

**Table 3:** Relative risk of positive psychotic-like symptoms in relation to estimated dietary intake of fatty acids

Dietary intake g/day·MJ		Positive psychotic-like symptoms ^a^											
		
Median	Interquintile range	Low level group		Middle level group					High level group				
					**Energy adjusted**		**Multivariate^b^**			**Energy adjusted**		**Multivariate^b^**	

**Marine fatty acids^c^**		**No.**		**No.**	**RR**	**95% CI**	**RR**	**95% CI**	**No.**	**RR**	**95% CI**	**RR**	**95% CI**

0.016	(0.00-0.02)	4478	Ref.	3637					237				
0.030	(0.02-0.04)	4606		3587	0.94	0.89-1.00	0.97 ^d^	0.91-1.03	177	0.71	0.58-0.87	0.75 ^d^	0.61-0.92
0.050	(0.04-0.06)	4733		3538	0.91	0.86-0.97	0.96 ^d^	0.90-1.02	176	0.69	0.57-0.85	0.76 ^d^	0.61-0.93
0.070	(0.06-0.80)	4594		3633	1.00	0.94-1.06	1.05 ^d^	0.98-1.13	227	0.96	0.80-1.16	1.05 ^d^	0.85-1.29
**Omega-6 fatty acids^e^**													
0.6	(0.1-0.7)	4408	Ref.	3712					261				
0.7	(0.6-0.8)	4709		3513	0.88	0.82-0.93	0.88 ^f^	0.83-0.94	182	0.64	0.53-0.78	0.67 ^f^	0.55-0.82
0.8	(0.7-0.8)	4670		3582	0.90	0.85-0.96	0.91 ^f^	0.86-0.97	173	0.62	0.51-0.75	0.66 ^f^	0.54-0.81
0.9	(0.9-2.1)	4624		3588	0.92	0.86-0.98	0.93 ^f^	0.86-0.99	201	0.73	0.60-0.88	0.78 ^f^	0.64-0.97
**Omega-3 fatty acids^g^**													
0.14	(0.02-0.17)	4444	Ref.	3663					252				
0.18	(0.17-0.20)	4613		3628	0.95	0.89-1.01	0.98 ^b^	0.92-1.04	175	0.66	0.54-0.81	0.73 ^h^	0.60-0.89
0.21	(0.20-0.22)	4736		3512	0.90	0.84-0.96	0.95 ^b^	0.89-1.01	176	0.66	0.53-0.80	0.76 ^h^	0.62-0.94
0.26	(0.22-0.93)	4618		3592	0.96	0.90-1.02	1.02 ^b^	0.95-1.09	214	0.83	0.69-1.00	1.00 ^h^	0.82-1.23

^a ^Participants categorized into levels of psychotic-like symptoms; low, middle, high (see methods section)

^b ^In the multivariate analysis presented, we initially tested the effects of adjusting also for BMI, level of education, country of birth, smoking, dietary intake of meat, dairy products, fruits, cereals and refined sugar, rheumatoid arthritis, gluten intolerance, diabetes and multivitamin supplement. However, none of these covariates change the estimates substantially, and was therefore not included in the final multivariate model

^c ^Sum of eicosapentaenoic acid (EPA), docosahexaenoic acid (DHA) and docosapentaenoic acid

^d ^Adjusted for age, total energy intake and dietary intake of vitamin B12, alcohol and dietary intake of fat other than eicosapentaenoic acid, docosahexaenoic acid and docosapentaenoic acid

^e ^Sum of arachidonic and linoleic acids

^f ^Adjusted for age, total energy intake and dietary intake of vitamin B12, alcohol and dietary intake of other than omega-6 fatty acids

^g ^Sum of eicosapentaenoic acid (EPA), docosahexaenoic acid (DHA), docosapentaenoic acid and α-linolenic acid

^h^Adjusted for age, total energy intake and dietary intake of vitamin B12, alcohol and dietary intake of other than omega-3 fatty acids

The intake of vitamin D was significantly associated with a decreased relative risk of both the middle and high levels of psychotic-like symptoms (Table 4). For example, after multivariate adjustment, the risk of middle respective high level psychotic-like symptoms for intake of vitamin D were respectively 18% and 37% lower in the highest quartile compared to the lowest quartile of intake.

**Table 4:** Relative risk of positive psychotic-like symptoms in relation to estimated dietary intake of vitamin D

Dietary intake g/day·MJ		Positive psychotic-like symptoms ^a^											
		
Median	Interquintile range	Low level group		Middle level group					High level group				
					**Energy adjusted**		**Multivariate^b^**			**Energy adjusted**		**Multivariate^b^**	

**Vitamin D**		**No.**		**No.**	**RR**	**95% CI**	**RR**	**95% CI**	**No.**	**RR**	**95% CI**	**RR**	**95% CI**

0.4	(0.01-0.5)	4132	Ref.	3668					240				
0.6	(0.5-0.6)	4476		3462	0.87	0.82-0.93	0.88 ^a^	0.82-0.93	185	0.71	0.58-0.86	0.72 ^a^	0.59-0.88
0.7	(0.6-0.8)	4590		3393	0.84	0.79-0.89	0.84 ^a^	0.79-0.90	171	0.64	0.53-0.79	0.65 ^a^	0.52-0.80
0.9	(0.8-4.0)	4620		3343	0.83	0.78-0.88	0.82 ^a^	0.77-0.88	175	0.66	0.54-0.81	0.63 ^a^	0.50-0.78

^a ^Adjusted for age, total energy intake, country of birth, BMI and dietary intake of vitamin B12

## Discussion

We report here results from the first study that has evaluated the dietary intake of fish, PUFA and vitamin D in relation to the experience of positive psychotic-like symptoms in a large cohort of over 30 000 Swedish women. We found support for a protective effect regarding the risk of positive psychotic-like symptoms with high dietary intake of fish, omega-3 and omega-6 PUFA, as well as of vitamin D. The associations were J-shaped with the strongest reduced risk for an intermediate intake of fish or PUFA.

Our findings provide further support for the hypothesis that an aberration in lipid metabolism may be involved in the biochemical basis for psychiatric disorders [13]. This suggestion has earlier been supported by ecological studies showing that variations in schizophrenia outcome between countries may be due to differences in the diet [6,14]. Further evidence for the hypothesis that PUFA is involved in the etiology of psychiatric disease has been put forward through the study by Stokes et al. showing a negative correlation between dietary PUFA intake and the severity of psychotic symptoms [15], as well as studies showing that patients with schizophrenia or depression have lower levels of PUFAs in brain tissue, red blood cells and skin fibroblasts and with a low intake of fish and PUFAs [16,29-31]. Finally, supplementation of PUFA, especially EPA, has been reported to be of possible benefit for patients with schizophrenia [17].

No earlier studies that we are aware of have evaluated the relationship between adult vitamin D levels and psychotic symptoms. Our findings indicated a protective effect of vitamin D for the risk of psychotic-like symptoms. Prenatal vitamin D deficiency has been proposed to be a risk factor for the development of schizophrenia [12]. Results from the Northern Finland 1966 Birth Cohort showed that vitamin D supplementation during the first year of life was associated with a reduced risk of schizophrenia in males, but not in women [32]. However, a small pilot study of maternal vitamin D levels in archived prenatal sera, showed no decrease in prenatal vitamin D in subjects who later developed schizophrenia [33]. It has been hypothesised that vitamin D insufficiency could account for some of the increased risk of schizophrenia observed among dark-skinned immigrants moving to countries with less sun exposure [12]. Vitamin D is to a large extent metabolized in the body through sun exposure, and people with dark skin need more sun exposure to maintain adequate blood levels. Our results of a protective effect of vitamin D intake must be considered as rough, since we only measure the dietary intake of vitamin D. A more complete picture of the vitamin D status could have been supplied through vitamin D levels in blood; however, no biological samples are available. The absorption of dietary vitamin D is generally high at all stages of life [34], but serum levels also depend on the endogenous production of vitamin D due to sun exposure which is subject to seasonal variations. Thus, the correlation between vitamin D intake and serum levels may vary. However, Burgaz et al. recently reported that 2-3 weekly servings of fatty fish increased 25(OH)D by 45% in a population of Swedish women [35]. Our results of the protective effect of fatty fish could in part be due to the content of vitamin D. While the epidemiological evidence linking low prenatal vitamin D and schizophrenia remains inconclusive, rodent models have provided compelling evidence about the role of vitamin D deficiency for brain development like larger lateral volumes [36], subtle memory dysfunction and altered attention processing [37] which have implications for neuropsychiatric disorders.

Unexpectedly, the intake of fatty fish (salmon, herring and mackerel) or shellfish more than twice a week increased the risk of being in the group with the highest level of psychotic-like symptoms. This puzzling finding may be due to unknown or known unhealthy constituents of fatty fish. For instance, environmental pollutants such as polychlorinated biphenyls (PCB) and dioxins are known to accumulate in fatty fish [38]. Another possible explanation may be that the frequent intake of fish and PUFA may be advantageous in lower doses but disadvantageous in higher doses. Reports by Mischoulon et al. and Peet & Horrobin suggest that there might be such a therapeutic window for DHA and EPA regarding their protective role for schizophrenia or depression [39-41]. Significant higher levels of DHA have been found in red cell membranes of un-medicated schizophrenic patients compared to healthy control subjects [42]. Furthermore, our findings of a more pronounced protective effect of omega-6 fatty acids than for omega-3 fatty acid are in agreement with the results from an EPA supplementation study in schizophrenia presented by Horrobins et al. [43]. This study surprisingly found that the effect of intermediate doses of EPA increased the levels of AA (an omega-6 PUFA) in the membrane of red cells, whereas higher doses of EPA did not, and the EPA-induced rise in AA was associated with a clinical improvement. The respective biological effects of omega-3 fatty acids and omega-6 fatty acids on the etiology of psychiatric symptoms could very well differ and the balance between the intakes of these fatty acids might be of importance. A high intake ratio of omega-3:omega-6 fatty acids favor omega-3 fatty acid metabolism. For example, high intake of omega-3 fatty acids partly replaces omega-6 fatty acids incorporation into membrane phospholipids and omega-3 fatty acids have a higher affinity than omega-6 fatty acids for several enzymes [44]. It has been proposed that the ratio of omega-3:omega-6 fatty acids might be more important in inhibiting the development of several diseases, including cancer, inflammatory and heart diseases [45,46]. We find no support for this in our results, since, the effect of omega-3:omega-6 fatty acids on psychotic-like symptoms were almost similar to those of omega-3 fatty acids. However, the mechanisms of action and protective abilities of PUFA could differ between different diseases.

The non-linearity in the association might seemingly argue against a possible causal relationship. However, the associations with dietary components and health are often non-linear with advantageous effects of a balanced nutrition [47-50]. We have no baseline measure of symptom levels to further elucidate causality between different levels of intake of fatty fish or shellfish and the risk of positive psychotic-like symptoms. However, keeping the prevalence figures for psychotic disorders in mind, rather few participants in our population sample are likely to have a disorder, which may diminish the problem of reversed causality related to psychotic diagnosis or medication.

In our study, the definition of psychotic-like symptoms was based on self-reported frequency of psychosis-like experiences. The classification of women into three groups with different levels of symptoms (low, middle and high) was based on predefined, but arbitrary cut-offs from the self-reported answers to the CAPE questionnaire. We acknowledge the limitation that the scale have not been used earlier in Sweden and validated in the Swedish population. Among women in the group with the highest level of psychotic-like symptoms, overweight, obesity and smoking were more common, and this group also contained more women who had migrated to Sweden. These characteristics are often seen in patients with psychotic disorders [51-53]. Based on the sum population prevalence of schizophrenia and other psychoses in middle-aged women [54], we might expect that 2-3% of the study participants would cross the boundaries to clinically valid syndromes. Our high level symptoms group consisted of 840 persons or about 2.5% of the whole study group. The CAPE measures of psychosis are strongly correlated with measures of general psychopathology, including depression. The association between the positive and the depressive dimension in CAPE, which we unfortunately could not include for practical reasons, is fairly low when distress associated with positive symptoms is held constant (r = 0.25; Stefanis et al., 2002 [1]). Thus, there are reasons to believe that the dimension of positive symptoms is an independent dimension.

In terms of generalisability of our results it is relevant to compare the levels of dietary intake of PUFAs and vitamin D in our cohort to other populations. The contribution of dietary intakes of PUFAs to total dietary fat is comparable to other Western cohorts [55]. The quotient omega-6 PUFA/omega-3 PUFA was 4:1 in this study, and this value is comparable to other European cohorts [56,57], but lower than that has been reported for US populations (Food and Nutrition Board. Dietary Reference Intakes for macronutrients Institute of Medicine, National Academic Press, Washington: 2005). The dietary intake of vitamin D in this cohort is comparable to several other European populations [58]. It is relevant to note that in Sweden low-fat dairy products and margarines are fortified with vitamin D. Otherwise the dietary intake of vitamin D would be lower. Furthermore, men were not included in the study and there is gender difference in the prevalence of psychosis [59]. However psychotic-like experiences in the general population might be more equally distributed among women and men [60].

The strengths of our study include its thorough diet data design and large sample size. The ethnic homogeneity of our study population reduces the risk of confounding by unmeasured factors, both genetic and environmental. We were able to adjust for smoking, BMI, migration, education and alcohol that could confound our associations between diet and psychotic-like symptoms. We had no information about socioeconomic status, but the adjustment for education, which is strongly associated with socioeconomic status, did not change the estimates. Still, we cannot rule out that there are unknown confounders that we have not been adjusted for, for example drugs influencing levels of serum lipids or family history of psychiatric illness. If the proportion of non-urbanized participants was high in the study population this could have confounded our results, nevertheless this is not the case in the Swedish population. Misclassification of fish and PUFA or vitamin D intake due to measurement error associated with the food frequency questionnaire is unavoidable, but given the study design likely non-differential, and thus attenuating any true association. It is also relevant to note that Hibbeln et al. reported high correlations (r = 0.7) between the dietary intake of EPA and DHA and serum levels of EPA and DHA in subjects with schizophrenia [51], indicating a good ability to report dietary intake accurately. An important limitation of this study is that we measured dietary intake only once, involving misclassification among those who changed their dietary pattern during follow-up. However, again some misclassification of dietary intake cannot be ruled out, such a misclassification is likely to be non-differential, and attenuating any true association. Unfortunately, we do not have information about specific vitamin D supplementation. But, adjustment for multivitamin supplement intake, containing vitamin D, did not change the estimates. None of the women in this study reported the use of dietary supplements containing fish oil or PUFA at baseline. Unfortunately, we do not have any information about the use of such supplements during follow-up. However, according to national figures from the National Food Administration, a low number of Swedish women took fish oil supplements (1%) at the time of the study.

We compared a number of characteristics for participants who completed the questionnaire, both in the parent study and the follow-up study, with those who only answered the questionnaire in the parent study. Age and overall dietary intake as well as the intake of fish and fatty acids did not differ significantly between those who participated in the follow-up study and those who did not (drop-outs). The drop-outs had a slightly higher BMI, lower education and were more often smokers and born outside of the Nordic countries. However, our main exposure (fish/fatty acids) did not differ between those who participated and those who did not participate in the study.

## Conclusions

In conclusion, in this large-scale cohort of Swedish women we found that a frequent consumption of fish, omega-3 and omega-6 fatty acids appears to reduce the risk of positive psychotic-like symptoms. The associations for fatty fish or shellfish were J-shaped, with the strongest inverse association for an intermediate dietary intake, whereas a high intake was associated with a higher rate of high level psychotic-like symptoms. Interestingly, we found a lower rate of psychotic-like symptoms with increasing vitamin D intake. Future studies are warranted for replication and should evaluate if this is a causal relationship. There are no current studies that can give real good answers to the question if dietary deficiency interacts with genetic vulnerability for schizophrenia. Schizophrenia is a multifactorial disorder with strong genetic vulnerability and the vulnerability might include metabolic aberrations. Further, it would be of interest to study if dietary aspects are associated with separable latent dimensions of positive psychotic-like symptoms and if the associations are valid for men and for other age groups.

## Abbreviations

AA: arachidonic acid; BMI: body mass index; CAPE: community assessment of psychic experiences; CI: confidence intervals; DHA: docosahexaenoic acid; DPA: docosapentaenoic acid; EPA: eicosapentaenoic acid; FFQ: food-frequency questionnaire; PCB: polychlorinated biphenyls; PUFA: polyunsaturated fatty acids; RR: relative risk; SD: standard deviation.

## Competing interests

The authors declare that they have no competing interests.

## Authors' contributions

EW was responsible for the recruitment, data collection and funding. CMH was responsible for the questions on positive psychotic-like symptom, funding and the initial idea in collaboration with MH. MH processed all food diaries and calculated intake of energy and nutrients, prepared a database with all variables as well as performed the data analyses, in collaboration with MOl who supervised the statistical analyses. TL and BN contributed with specific knowledge in the topic of fatty acids and psychiatric diseases. MH prepared the manuscript in collaboration with ML. All authors have interpreted the results, reviewed and approved the final manuscript.

## Pre-publication history

The pre-publication history for this paper can be accessed here:

http://www.biomedcentral.com/1471-244X/10/38/prepub

## Supplementary Material

Additional file 1 **Appendix 1**. Questions on positive psychotic-like symptoms (Community Assessment of Psychic Experiences, CAPE-42, Stefanis et al., 2002 [1]), answered by 33 623 women in the follow-up study of Women's Lifestyle and Health Cohort Click here for file
