# Supplementary material for: Dietary intake of fish, omega-3, omega-6 polyunsaturated fatty acids and vitamin D and the prevalence of psychotic-like symptoms in a cohort of 33 000 women from the general population
Source: BMC Psychiatry. 2010 May 26;10:38. doi: 10.1186/1471-244X-10-38 (PMC2889879; doi:10.1186/1471-244X-10-38)
Supplement: Additional file 1 — Appendix 1. Questions on positive psychotic-like symptoms (Community Assessment of Psychic Experiences, CAPE-42, Stefanis et al., 2002 [1]), answered by 33 623 women in the follow-up study of Women's Lifestyle and Health Cohort [file 1471-244X-10-38-S1.DOC]

**Appendix 1 - Questions on positive psychotic-like symptoms (Community Assessment of Psychic Experiences, CAPE-42, Stefanis et al., 2002 [1]), answered by 33 623 women in the follow-up study of Women's Lifestyle and Health Cohort**

| **Questions** | | **Answer alternatives**  **(frequency, in %)** | | | | |
| --- | --- | --- | --- | --- | --- | --- |
|  |  | **Almost always** | **Often** | **Sometimes** | | **Never** |
| 1. | Do you ever feel as if people seem to drop hints about you or say things with a double meaning? | 0.3 | 1.8 | | 40 | 57 |
| 2. | Do you ever feel as if things written in magazines or on TV are especially for you? | 0.3 | 0.3 | | 12 | 87 |
| 3. | Do you ever feel as if some people are not what they seem to be? | 0.2 | 5.2 | | 62 | 33 |
| 4. | Do you ever feel as if you are being persecuted in some way? | 0.2 | 0.2 | | 3.9 | 96 |
| 5. | Do you ever feel as if there is a conspiracy against you? | 0.2 | 0.3 | | 7.5 | 92 |
| 6. | Do you ever feel as if you are destined to be someone very important? | 0.8 | 3.7 | | 24 | 72 |
| 7. | Do you ever feel that you are a very special or unusual person? | 0.9 | 4.0 | | 34 | 61 |
| 8. | Do you ever think that people can communicate telepathically? | 1.2 | 5.8 | | 46 | 47 |
| 9. | Do you ever feel as if electrical devices such as computers can influence the way you think? | 0.2 | 0.4 | | 4.4 | 95 |
| 10. | Do you believe in the power of witchcraft, voodoo or the occult? | 1.3 | 2.1 | | 18 | 79 |
| 11. | Do you ever feel that people look at you oddly because of your appearance? | 0.4 | 1.0 | | 14 | 85 |
| 12. | Do you ever feel as if the thoughts in your head are being taken away from you? | 0.2 | 0.4 | | 5.6 | 94 |
| 13. | Do you ever feel as if the thoughts in your head are not your own? | 0.2 | 0.3 | | 5.3 | 94 |
| 14. | Have your thoughts ever been so vivid that you were worried other people would hear them? | 0.2 | 0.2 | | 4.4 | 95 |
| 15. | Do you ever hear your own thoughts being echoed back to you? | 0.2 | 0.3 | | 5.5 | 94 |
| 16. | Do you ever feel as if you are under the control of some force or power other than yourself? | 0.3 | 0.4 | | 4.2 | 95 |
| 17. | Do you ever hear voices when you are alone? | 0.1 | 0.1 | | 2.2 | 98 |
| 18. | Do you ever hear voices talking to each other when you are alone? | 0.1 | 0.05 | | 0.5 | 99 |
| 19. | Do you ever feel as if a double has taken the place of a family member, friend, or acquaintance? | 0.2 | 0.1 | | 1.3 | 98 |
| 20. | Do you ever see objects, people or animals that other people cannot see? | 0.2 | 0.4 | | 3.8 | 96 |
